# Maternal and neonatal health in Canada’s Black communities: A scoping review of epidemiologic studies

**DOI:** 10.17269/s41997-025-01102-9

**Published:** 2025-09-04

**Authors:** Ebonee Lennord, Elsie Amoako, Maya Rajasingham, Abirami Kirubarajan, Rohan D’Souza, Isabelle Malhamé, Susie Dzakpasu, Modupe Tunde-Byass, Cynthia Maxwell, Giulia M. Muraca

**Affiliations:** 1 Department of Obstetrics & Gynecology, McMaster University, Hamilton, ON Canada; 2 Mino Care, Toronto, ON Canada; 3 Department of Health Research Methods, Evidence, and Impact, McMaster University, Hamilton, ON Canada; 4 Department of Medicine, McGill University Health Centre, Montréal, QC Canada; 5 Centre for Outcomes Research and Evaluation, Research Institute of the McGill University Health Centre, Montréal, QC Canada; 6 Maternal and Infant Health Section, Public Health Agency of Canada, Ottawa, ON Canada; 7 Department of Obstetrics and Gynaecology, University of Toronto, Toronto, ON Canada; 8 Department of Obstetrics and Gynecology, North York General Hospital, Toronto, ON Canada; 9 Department of Obstetrics and Gynaecology, Women’s College Hospital and Sinai Health, Toronto, ON Canada

**Keywords:** Maternal health, Infant, Newborn health, Health equity, Black or African American, Racial groups, Epidemiologic studies, Santé maternelle, Santé néonatale, Équité en santé, Noirs/Afro-Américains, Groupes raciaux, Études épidémiologiques

## Abstract

**Objectives:**

Black-White disparities in maternal and neonatal morbidity and mortality highlight health inequities in several settings, yet such racial disparities in Canada are not well defined. Our objective was to conduct a scoping review to identify the extent of epidemiologic evidence assessing Black-White disparities in maternal and neonatal health in Canada.

**Methods:**

We included peer-reviewed epidemiologic studies which measured maternal or neonatal outcomes in Black versus White individuals in Canada. We searched OVID platforms (MEDLINE, Embase, Emcare) from inception to May 9, 2024, using keywords and controlled vocabulary terms related to race and maternal and neonatal morbidity and mortality. Results synthesis was carried out using descriptive analysis.

**Synthesis:**

After exclusions, six retrospective cohort studies were included in the scoping review. The majority of the included studies used data obtained from provincial datasets (*n* = 5), defined maternal race using self-reported race (*n* = 5), and were set in Ontario (*n* = 4). All studies reported one or more significant associations between race and adverse maternal or neonatal outcomes, with Black individuals experiencing higher rates of spontaneous fetal loss (*n* = 1), perinatal mortality (*n* = 1), preterm birth (*n* = 3), small for gestational age infants (*n* = 1), low Apgar scores (*n* = 2), congenital heart disease (*n* = 1), neonatal intensive care unit admission (*n* = 1), preeclampsia (*n* = 2), gestational diabetes (*n* = 1), and inadequate gestational weight gain (*n* = 1).

**Conclusion:**

Although literature on the topic is sparse, Black-White disparities in maternal and neonatal health in Canada are apparent. National, population-based data are needed to provide a comprehensive understanding of racial disparities in maternal and neonatal health and the factors driving these differences.

**Supplementary Information:**

The online version contains supplementary material available at 10.17269/s41997-025-01102-9.

## Résumé

**Objectif:**

Les disparités entre les personnes noires et blanches en matière de morbidité et de mortalité maternelle et néonatale mettent en évidence des inégalités en santé dans plusieurs contextes, mais ces disparités raciales au Canada ne sont pas bien définies. Nous avons mené une revue de la portée pour déterminer l'étendue des données épidémiologiques évaluant les disparités entre les personnes noires et blanches en matière de santé maternelle et néonatale au Canada.

**Méthodes:**

Nous avons inclus des études épidémiologiques évaluées par des pairs qui comprenant des données aux niveaux maternel ou néonatal chez les personnes noires et blanches au Canada. Nous avons effectué des recherches sur les plateformes OVID (MEDLINE, Embase, Emcare) depuis leur création jusqu'au 9 mai 2024, à l'aide de mots clés et de termes de vocabulaire contrôlé liés à la race et à la morbidité et la mortalité maternelles et néonatales. La synthèse des résultats a été réalisée par analyse descriptive.

**Synthèse:**

Après exclusions, six études de cohorte rétrospectives ont été incluses dans la revue. La majorité des études incluses utilisaient des données provinciales (n = 5), définissaient l'origine ethnique maternelle à partir de l'origine ethnique autodéclarée (n = 5) et se déroulaient en Ontario (n = 4). Toutes les études ont signalé une ou plusieurs associations significatives entre la race et les issues maternelles ou néonatales défavorables, les personnes noires présentant des taux plus élevés de pertes fœtales spontanées (n = 1), de mortalité périnatale (n = 1), de naissances prématurées (n = 3), de petits poids pour l’âge gestationnel (n = 1), de score d'Apgar faible (n = 2), de cardiopathie congénitale (n = 1), d’admissions à l’unité de soins intensifs néonataux (n = 1), de prééclampsie (n = 2), de diabète gestationnel (n = 1) et de prise de poids gestationnelle inadéquate (n = 1).

**Conclusion:**

Malgré la rareté des publications sur le sujet, les disparités entre les personnes noires et blanches en matière de santé maternelle et néonatale au Canada sont évidentes. Des données nationales populationnelles sont nécessaires pour comprendre en profondeur les disparités raciales en matière de santé maternelle et néonatale et les facteurs qui les expliquent.

## Introduction

Racial inequities in maternal and neonatal health are pervasive in high-income countries and remain a major source of health inequities in many settings. In particular, Black-White disparities in maternal and neonatal mortality persistently highlight health inequities in countries such as the United States of America (US) (Ely & Driscoll, 2024; Hoyert, 2024; Joseph et al., 2021; Louis et al., 2015) and the United Kingdom (UK) (Felker et al., 2024; Knight et al., 2023; Odd et al., 2024). Recent evidence from Europe, Australia, and the US has shown similar trends in severe maternal and neonatal morbidity, demonstrating Black-White disparities in severe pregnancy-related illness, prolonged hospitalization, and long-term disability (Adane et al., 2020; Admon et al., 2018; Creanga et al., 2014a, 2014b; Kramer et al., 2006; Leonard et al., 2019; Somer et al., 2017; Thompson & Suter, 2020; Zwart et al., 2011).

Significant Black-White disparities in maternal and neonatal mortality have been demonstrated in several studies. For example, maternal mortality rates are 2.5- to threefold higher among Black versus White individuals in the US, the UK, and Brazil (Felker et al., 2024; Hoyert, 2024; Joseph et al., 2021; Silva et al., 2024). Similarly, 1.5- to 2.5-fold higher rates of severe maternal morbidity, a composite measure of severe pregnancy-related complications, have been shown among Black versus White pregnant individuals in Australia, the Netherlands, the UK, and the US (Adane et al., 2020; Admon et al., 2018; Felker et al., 2024; Leonard et al., 2019; Zwart et al., 2011). In addition, White pregnant people are less likely to experience hypertensive disorders (Bryant et al., 2005; Tanaka et al., 2007) and puerperal infection (Guendelman et al., 2006) than Black pregnant people, and these differences persist after accounting for individual risk factors and traditional measures of socioeconomic status (SES) (Bailey et al., 2021; Leonard et al., 2019).

Regarding neonatal outcomes, several studies have also documented stark differences in Black-White morbidity and mortality. For example, in the US, more than twofold higher rates of infant death, preterm birth, and low birth weight were shown among infants born to non-Hispanic Black people compared with non-Hispanic White people (Ely & Driscoll, 2024; MacDorman, 2011; Matoba & Collins Jr, 2017; Schempf et al., 2007; Sparks, 2009; Willinger et al., 2009). The same patterns have been shown with higher rates of intrauterine growth restriction, low Apgar score at birth (5-min Apgar < 4 and < 7), and stillbirth in Black vs White groups (Frisbie et al., 1997; Healy et al., 2006; Kramer et al., 2006; Sparks, 2009).

In Canada, evidence regarding the relationship between race and maternal and neonatal health is not well defined. This limitation has recently come under scrutiny as the COVID-19 pandemic magnified the unmet need for robust data to recognize and respond to the health inequities experienced by racialized groups in Canada (Adhopia, 2021; Andrew-Gee & Grant, 2019; Canadian Institute for Health Information, 2020; Datta et al., 2021; Dryden & Nnorom, 2021; Grant et al., 2019; Thompson et al., 2021). Several features of the history and politics of race relations in Canada make it distinct from experiences in other countries and preclude the extrapolation of evidence to the Canadian population (Attewell et al., 2010; Frazier et al., 2010). Canada’s history, politics, immigration patterns, and healthcare delivery model limit the generalizability of other countries’ maternal/neonatal experiences to the Canadian context (Frazier et al., 2010; Statistics Canada, 2019).

We conducted a scoping review to understand the state of the currently available literature on the topic of Black maternal and neonatal health in Canada.

## Methods

This review was conducted and reported in accordance with the Joanna Briggs Institute (Aromataris et al., 2024) framework for scoping reviews and the PRISMA Extension for Scoping Reviews (Tricco et al., 2018). The protocol was registered on the Open Science Framework (Muraca, 2025). The following questions guided the scoping review:

1. What are the disparities in maternal and neonatal mortality and severe maternal and neonatal morbidity between Black and White pregnant people?
2. What are the proposed mechanisms to explain any observed disparities?
3. What are the identified knowledge gaps in the available literature?

### Inclusion criteria

#### Population

Black and White pregnant individuals and their offspring.

#### Concept

What are the comparative maternal and perinatal health indicators in Black and White populations in Canada?

#### Context

We reviewed evidence that supports the context of Black individuals living in Canada. We excluded studies that were not based in Canada and those that did not compare maternal/neonatal outcomes in Black vs White individuals (groups must have included a direct maternal race measure (Black/White), not a race proxy (e.g., maternal country of birth). The context of this review was grounded in Critical Race Theory (Crenshaw et al., 1995) and intentionally restricted to Black and White individuals to align with our central goal of understanding the health impacts of racism, and specifically anti-Black racism, within a context of structural inequality. Black populations in North America have historically faced—and continue to face—disproportionate exposure to structural, institutional, and interpersonal racism across nearly every domain of life, including healthcare. In contrast, White individuals, as the group considered as having the most social power and the least exposure to racism in this framework, provide a reference point that makes it possible to examine the effects of racialization with greater conceptual clarity. While we acknowledge the importance of studying the experiences of other racialized groups, the mechanisms, manifestations, and consequences of racism differ across populations. This focused analysis allows us to examine anti-Black racism specifically, rather than conflating it with other forms of racial marginalization that may have distinct historical and sociopolitical roots.

#### Types of sources

We considered observational epidemiologic studies (e.g., cohort, cross-sectional). We excluded case reports, case series, and conference abstracts.

### Search strategy

The search strategy was developed with an information scientist and was in line with PRISMA guidelines for scoping reviews. We searched the OVID database including MEDLINE, Embase, and Emcare using keywords and controlled vocabulary terms (e.g., “Black”, “maternal”, “perinatal”, “pregnancy”, “Canada”, “racial disparities”, “racism”, “severe maternal morbidity”, “maternal mortality”, “perinatal morbidity”) from inception to May 9, 2024 (Appendices 1–3). We had no restrictions based on year of publication or language. Additional papers were included through hand searching references of included papers. The search results were managed in Covidence (Veritas Health Innovation, 2024).

### Study selection

Two reviewers (EL and GMM) independently screened titles and abstracts of the articles retrieved from the search for study eligibility. We conducted content analysis of included studies to thoroughly examine if/how the three research questions were addressed. Disagreements were resolved through discussions with both reviewers. If disagreements persisted, conflicts were raised until a consensus was reached. Articles deemed potentially eligible were carried forward for full-text screening by the two reviewers, independently using Covidence to select the final articles using the predefined inclusion and exclusion criteria.

### Data extraction

We extracted study characteristics including title, last name of first author, year of publication, population studied, number of individuals included, outcome(s) of interest, race ascertainment (measure), study design, data source, as well as crude and adjusted outcome rates in Black vs White individuals (Table 1).

**Table 1:** Studies of maternal and perinatal outcomes in Black vs White individuals in Canada

Author(s) Year	Outcome(s) of interest	Population Number of individuals included (*B*, Black; *W*, White)	Race ascertainment	Study design Data source(s)	Crude rate of maternal/perinatal morbidity/mortality outcomes in Black vs White individuals	Adjusted odds/risk ratio (95% confidence interval) for Black vs White (reference) grp
(Rey, 1997)	Preeclampsia, perinatal mortality, preterm birth, small for gestational age (SGA) infant, Apgar < 7 at 1 and 10 min, induction of labor, cesarean delivery	Pregnant individuals (Black—born in Haiti and White—born in Canada) with mild chronic hypertension followed and delivered at Sainte-Justine Hospital, Quebec (Jan 1987–Dec 1991) B: 74; W: 208	Self-reported race and recorded by the prenatal care provider	Retrospective analysis of longitudinal cohort Sainte-Justine Hospital records	Preeclampsia: 32.43% vs 14.90%	3.5 (1.6–7.6)
					Perinatal mortality: 9.46% vs 2.88%	3.8 (1.1, 15.2)
					SGA infant: 18.92% vs 12.50%	1.8 (0.7–4.2)
					Preterm birth: 32.43% vs 19.71%	1.9 (1.1–3.5)*
					1-min Apgar score < 7: 32.43% vs 23.08%	1.5 (1.0–2.2)*
					10-min Apgar score < 7: 9.46% vs 3.37%	2.0 (1.1, 7.8)*
(Wyatt et al., 2005)	Spontaneous fetal loss	Pregnant individuals with singleton births who used the Ontario Maternal Serum Screening Program (Oct 1995–Sep 2000) B: 13,826; W: 160,567	Self-reported race and recorded by care providers on the prenatal screening requisition	Retrospective cohort Ontario Maternal Serum Screening Program and Canadian Institute of Health Information	Spontaneous fetal loss: 1.80% vs 0.47%	3.8 (3.3–4.4)*
(McKinnon et al., 2016)	Preterm birth (< 37 weeks gestation) Very preterm birth (< 32 weeks gestation)	Singleton, live births in non-Hispanic Black vs non-Hispanic White individuals in Canada and the US (May 2004–May 2006) B: 3,811; W: 87,234	Self-identified race from the 2006 census question on visible minority status	Retrospective cohort Linked live birth, infant death, and stillbirth database with 2006 Canadian census	Preterm birth: 8.9% vs 5.9%	1.60 (1.39–1.81)
					Very preterm birth 1.9% vs 0.7%	2.62 (1.83–3.41)
(Guo et al., 2019)	Inadequate and excessive gestational weight gain	Pregnant individuals (White, Asian, and Black) who accessed prenatal screening with a singleton birth in Ontario (Apr 2016–Mar 2017) B: 5,129; W: 47,626	Self-reported race and recorded by care providers on the prenatal screening requisition	Retrospective cohort Better Outcomes Registry & Network (BORN) Ontario	Inadequate weight gain: 25.0% vs 15.7%	1.29 (1.24–1.33)
					Excessive weight gain: 62.8% vs 54.7%	0.83 (0.80–0.86)
(Miao et al., 2022a, 2022b)	Congenital heart disease	Pregnant individuals with singleton births who accessed prenatal screening in Ontario (Apr 2012–Mar 2018) B: 35,258; W: 315,919	Self-reported race and recorded by care providers on the prenatal screening requisition	Retrospective cohort Linkage between (a) Better Outcomes Registry & Network (BORN) Ontario; (b) Discharge Abstract Database; (c) National Ambulatory Care Reporting System; (d) Canadian Postal Code Conversion File	Congenital heart disease 1.62% vs 1.17%	1.40 (1.27–1.54)
(Miao et al., 2022a, 2022b)	Gestational diabetes, gestational hypertension, preeclampsia, placenta previa, placental abruption, preterm birth, operative delivery, episiotomy, and obstetric anal sphincter injury Low birth weight, macrosomia, small for gestational age, large for gestational age, 5-min Apgar score (< 7, < 4), arterial cord pH ≤ 7.1, hyperbilirubinemia requiring treatment, and neonatal intensive care unit (NICU) admission	Black and White pregnant people who attended prenatal screening and had a singleton birth in Ontario (Apr 2012–Mar 2019) B: 41,776; 370,344	Self-reported race and recorded by care providers on the prenatal screening requisition	Retrospective cohort Better Outcomes Registry & Network (BORN) Ontario	Gestational diabetes: 6.36% vs 5.08%	1.08 (1.04–1.13)
					Preeclampsia: 4.32% vs 4.06%	1.10 (1.05–1.14)
					Cesarean delivery: 32.95% vs 28.44%	1.11 (1.09–1.12)
					Operative vaginal delivery: 6.32% vs 8.99%	0.83 (0.79–0.87)
					Episiotomy: 5.69% vs 9.02%	0.71 (0.67–0.76)
					Obstetric anal sphincter injury: 2.15% vs 3.39%	0.72 (0.64–0.81)
					Preterm birth: 8.86% vs 6.35	1.41 (1.37–1.44)
					Low birth weight: 8.03% vs 4.36%	1.96 (1.92–2.00)
					Macrosomia: 7.89% vs 12.03%	0.61 (0.57–0.64)
					Small for gestation age: 12.16% vs 7.46%	1.77 (1.74–1.80)
					Large for gestational age: 7.88% vs 11.17%	0.64 (0.60–0.67)
					5-min Apgar score < 7: 3.53% vs 2.33%	1.63 (1.57–1.69)
					5-min Apgar score < 4: 1.62% vs 0.72%	2.29 (2.20–2.38)
					Arterial cord pH ≤ 7.1: 5.15% vs 5.89%	0.93 (0.88–0.98)
					NICU admission: 14.13% vs 11.65	1.25 (1.22–1.27)
					Hyperbilirubinemia requiring treatment: 14.37% vs 12.09%	1.21 (1.19–1.24)

^*^Crude RR as no adjusted RR reported

### Data synthesis

We produced narrative summaries to accompany the tabulated results to describe how the results relate to the research questions of the review. Common characteristics of the included studies, trends in the literature, areas of concentration, and remaining knowledge gaps were identified. No formal risk-of-bias assessment or meta-analysis was conducted, consistent with scoping review methodology.

## Results

**Fig. 1:**
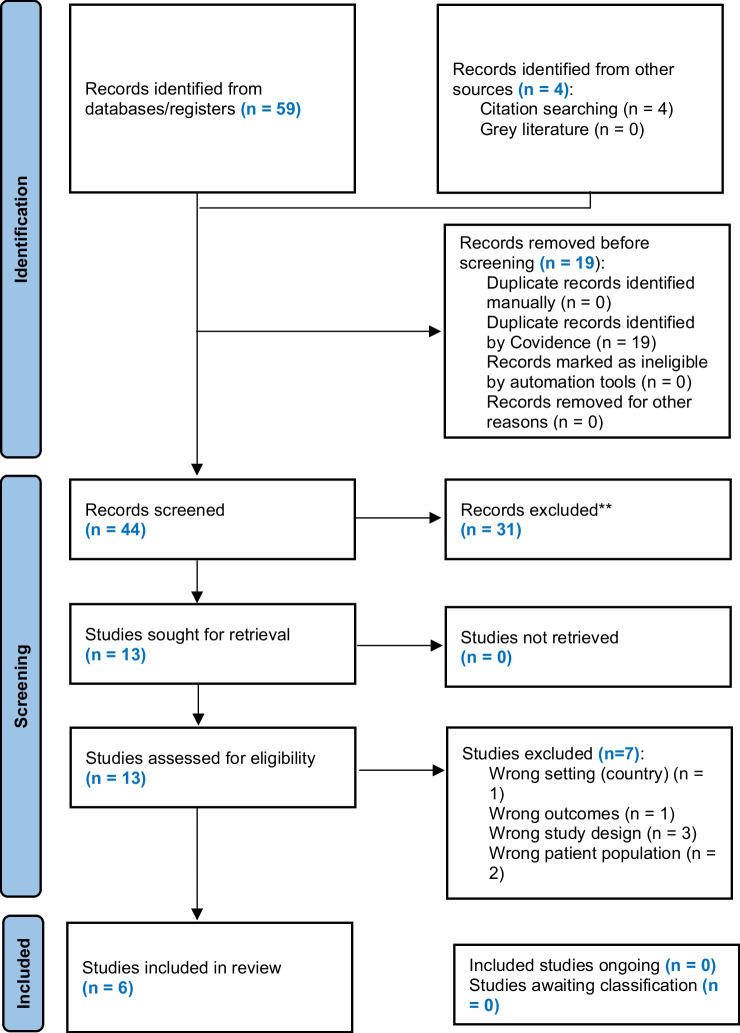
PRISMA diagram showing the derivation of studies included in review

A review of the literature identified six studies in Canada that compared maternal or neonatal outcomes between Black and White individuals (Fig. 1). All six studies were retrospective in design; one was a single-centre study in the province of Québec, one was a national study, and the remaining four were set in the province of Ontario (Table 1).

The earliest included study was a hospital-based analysis in Quebec of 74 Black and 208 White individuals with mild chronic hypertension between 1987 and 1991. These individuals were followed to compare rates of preeclampsia, perinatal mortality, preterm birth, small for gestational age infant, Apgar score < 7 at 1 and 10 min, induction of labour, and cesarean delivery. Maternal race was self-reported and recorded by the prenatal care provider. Outcomes were ascertained from the hospital admission records. Higher rates of preeclampsia (32.4% among Black individuals versus 14.9% among White individuals; adjusted RR 3.5, 95% CI 1.6–7.6) and perinatal mortality (9.5% vs 2.9% in Black vs White groups; adjusted RR 3.8, 95% CI 1.1–15.2) were shown. Crude rates of preterm birth, as well as 1- and 10-min Apgar scores < 7 were also found to be higher in Black versus White individuals (Table 1).

The single national study by McKinnon et al. (2016) compared Black-White disparities in preterm birth rates between Canada and the US using data from singleton live births (2004–2006). The data for the Canadian cohort included in this study were obtained through a linkage of Vital Statistics birth, stillbirth, and infant death records with the 2006 Long-Form Census records. Self-identified maternal race among Canadian individuals was identified from the census question on visible minority status, which asked the respondent to indicate whether they identified as: “White, Chinese, South Asian, Black, Filipino, Latin American, Southeast Asian, Arab, West Asian, Japanese, Korean, other (specify)?” Individuals who reported both “Black” and “White” were included in the “Black” group. The study included 91,045 births in Canada (3,811 among Black individuals and 87,234 among White individuals) and 5,069,267 in the US (1,039,642 and 4,029,625 among Black and White groups, respectively) and assessed both absolute and relative differences in preterm birth (< 37 weeks) and very preterm birth (< 32 weeks). In Canada, the preterm birth rate was 8.9% for Black individuals and 5.9% for White individuals, yielding a crude rate ratio (RR) of 1.49 (95% confidence interval [CI] 1.32–1.66) and risk difference of 2.94% (95% CI 1.91–3.96%). For very preterm birth, Canadian rates were 1.9% for Black versus 0.7% for White individuals (RR 2.70, 95% CI 1.95–3.44). Overall, the study showed the disparity in preterm birth between Black and White individuals in Canada to be similar in magnitude to those seen in the US (adjusted risk difference for preterm birth 3.59 [95% CI 2.32–4.85] in Canada and 3.57 [95% CI 3.43–3.70] in the US).

Four of the included studies (Guo et al., 2019; Miao et al., 2022a, 2022b; Wyatt et al., 2005) were population-based studies from Ontario examining Black-White disparities in maternal and neonatal outcomes among individuals who accessed prenatal genetic screening (~ 65 to 70% of the overall pregnant population) (Prenatal Screening Ontario, 2021). All four Ontario-based studies were restricted to this group since maternal race is not recorded in provincial health administrative or clinical datasets unless a pregnant person accessed prenatal genetic screening. In those who accessed prenatal genetic screening, self-reported maternal race was recorded on the screening requisition by care providers in pre-specified categories (Asian, Black, White, Other). Wyatt et al. (2005) compared spontaneous fetal loss occurring at or after 15 weeks of gestation among 13,826 Black and 160,567 White individuals with singleton pregnancies between 1995 and 2000. Spontaneous fetal loss was found in 1.80% and 0.47% in the Black and White groups, respectively (Odds ratio 3.8, 95% CI 3.3–4.4; Table 1).

Guo et al. (2019) conducted a study of racial variations in gestational weight gain among 5,129 Black and 47,626 White pregnant individuals with a singleton birth in Ontario (2012–2018). Data was obtained from the Better Outcomes Registry and Network (BORN) Ontario, a clinical registry containing information on 99% of births in Ontario. This study found that Black pregnancies had a higher risk of inadequate gestational weight gain and a lower risk of excessive gestational weight gain compared with White pregnancies. Specifically, inadequate weight gain was recorded in 25.0% of Black pregnancies compared with 15.7% of White pregnancies (adjusted RR 1.29, 95% CI 1.24–1.33; Table 1). Equivalent rates for excessive weight gain were 62.8% and 54.7% in Black and White pregnancies, respectively (adjusted RR 0.83, 95% CI 0.80–0.86).

The remaining two Ontario-based studies were both conducted by Miao et al., (2022a, 2022b). The first compared the incidence of congenital heart disease among 35,258 Black and 315,919 White pregnant individuals (Miao et al., 2022a). The study used a linkage of data from (1) the Better Outcomes Registry and Network (BORN) Ontario, a clinical registry of 99% of births in Ontario; (2) the Canadian Institute of Health Information (CIHI) Discharge Abstract Database, with information on hospital separations from all acute care centres in Ontario; (3) the CIHI National Ambulatory Care Reporting System, containing information on admissions to the emergency department, day surgery, and other ambulatory care visits; and (4) the Canadian Postal Code Conversion File, for neighbourhood-level indicators of income, education, and rurality. Congenital heart disease occurred in 1.62% of the Black group compared with 1.17% of the White group. The adjusted RR of congenital heart disease was 1.40 (95% CI 1.27–1.54) in Black vs White individuals, after controlling for neighbourhood-level household income, education, and rurality index, as well as maternal age, maternal obesity, assisted reproductive technology, smoking/drug use during pregnancy, mental health condition, maternal comorbidity, and infant sex.

The second study by Miao et al. (2022b) was the most in-depth evaluation of Black-White disparities in maternal and neonatal outcomes included in the review. This study included information on 412,120 singleton births; 41,776 and 370,344 among Black and White individuals, respectively. Outcomes of interest included several maternal and neonatal outcomes. Maternal outcomes comprised gestational diabetes, gestational hypertension, preeclampsia, placenta previa, placental abruption, operative delivery, episiotomy, and obstetric anal sphincter injury. Neonatal outcomes included low birth weight, macrosomia, small/large for gestational age, 5-min Apgar score < 7 and < 4, arterial cord pH ≤ 7.1, hyperbilirubinemia requiring treatment, and neonatal intensive care unit (NICU) admission. Data for this study was obtained by BORN Ontario. After adjustment for several maternal demographic, socioeconomic, and clinical factors, Black individuals had approximately 10% higher rates of gestational diabetes, preeclampsia, and cesarean delivery, and 15–30% lower rates of operative vaginal delivery (forceps/vacuum delivery), episiotomy, and obstetric anal sphincter injury (Table 1). With respect to neonatal outcomes, Black individuals had a 40% higher rate of preterm birth, a 77% higher rate of small for gestational age, and an almost twofold higher rate of low birth weight. Significantly higher rates of low Apgar score (< 7 and < 4) as well as hyperbilirubinemia requiring treatment and NICU admission were found among Black individuals (Guo et al., 2019; Miao et al., 2022a, 2022b; Rey, 1997; Wyatt et al., 2005).

## Discussion

This scoping review aimed to understand the state of the currently available literature on Black maternal and neonatal health in Canada. The results of our review show that only six studies have conducted epidemiologic analyses of Black-White disparities in maternal and neonatal outcomes in Canada. In terms of maternal outcomes, Black individuals had higher rates of preeclampsia, gestational diabetes, and inadequate gestational weight gain. With respect to neonatal outcomes, infants born to Black individuals were found to have higher rates of spontaneous fetal loss, perinatal mortality, preterm birth, small for gestational age infant, low Apgar score, congenital heart disease, and admission to the NICU.

Although the six identified studies address some knowledge gaps, they did not include individuals in several provinces and territories in Canada. Accessibility and quality of pregnancy care, racial demographics and immigration patterns vary among Canada’s provinces and territories, which confines the generalizability of the review results (4 of the 6 studies were Ontario-based) to the larger Canadian population. Further, the Ontario studies were restricted to individuals who accessed prenatal screening, which may bias findings if minoritized individuals are underrepresented in those who access prenatal screening.

Further, none of the included studies investigated potential structural, sociodemographic, or intersectional factors that may account for or drive differential rates of adverse outcomes in Black and White individuals. As such, the individual and structural forms of racism, their intersection/overlap with other social identities, and their relationship to maternal and neonatal health inequities remain uncertain. For example, gender, sexual identity, immigration status, disability, and other measures of social position were not considered, likely due to the paucity of information on health equity stratifiers in administrative and clinical databases in Canada. Identifying modifiable, systems-level indicators of structural racism (e.g., differences in maternity care provision or socioeconomic marginalization) is paramount for the design of interventions/policies to promote equity in maternal and neonatal health. Much work must be done to examine potential contributing factors, such as the role of economic security, neighbourhood and built environment, social community context, healthcare access, and lack of diversity in the pregnancy care workforce as well as intersectional effects of these factors.

Although not included in the review, studies that have used maternal birthplace and/or primary language to approximate race have similar findings. For example, two studies in Quebec comparing Haitian and non-Haitian-born individuals found higher rates of preterm birth, low birth weight, small for gestational age infants, and stillbirth (Auger et al., 2012, 2016). Another study comparing composite severe maternal morbidity rates by maternal birthplace found higher rates among pregnant people born in Sub-Saharan Africa and the Caribbean than among Canadian-born pregnant people (Urquia et al., 2017). While studies using proxies for race are informative of the experience of first-generation immigrants and their babies, these data cannot provide a complete or accurate picture of racial disparities in maternal and neonatal health across Canada.

The lack of routinely collected and reported race-based health data in Canada has made it challenging to quantify health inequities among Canadians. Appropriate and consistent collection of data on race in Canada’s population-based administrative health datasets and clinical registries would increase our understanding of the relationship between race and maternal and neonatal morbidity and mortality in Canada and has the potential to inform how social and healthcare policies are contributing to Black-White health inequities.

Race-disaggregated data has the power to reveal systemic inequalities and lead to policies that address these inequalities. In Canada, race-disaggregated data revealed higher rates of COVID-19 infection among racialized communities due to systemic inequalities in housing and employment (Thompson et al., 2021). This evidence guided policy adjustments such as prioritizing vaccine distribution in high-risk neighbourhoods to foster more equitable outcomes. By exposing inequities, targeted interventions could be developed to improve access, quality of care, and health outcomes. While such data can reveal systemic inequalities, there are also dangers of significant harm to individuals and communities. Data disaggregated by race has been used to perpetuate oppression, colonization, and systemic racism (Black Public Health Collective, 2020). Systematically marginalized communities have been the target of heightened surveillance and control measures, leading to further disenfranchisement and stigmatization. The case of the Toronto Police Service’s disproportionate force and enforcement actions against Black Canadians is an example of such a misuse of such data (Ontario Human Rights Commission, 2022).

With the appropriate use of race-disaggregated data, there is the potential to inform policies that can reduce health inequities. The data collection process must begin with the creation of an articulated plan for defining and analyzing race that is informed by community partners and underpinned by a commitment to dismantling interpersonal and systemic racism and oppression (Bailey et al., 2021; Krieger, 2014). Partnerships with racialized community advocacy groups can facilitate an understanding of the priorities, concerns, needs, and interests of racialized pregnant people as defined in the EGAP (Engagement, Governance, Access, Protection) Framework. These partnerships will be foundational to addressing the most pertinent needs of communities, crafting best practices in the collection, management, use, analysis, and dissemination of information. Engaging with community partners creates space for marginalized voices to be involved in policy development that addresses systemic inequalities without exacerbating health inequities. Finally, data that allows for the comparison of health outcomes between racially defined communities supports Canada’s human rights legal framework by interrogating the fundamental principle of equality in the Canadian Charter of Rights and Freedoms (1982). If we continue to use a “colour-blind” approach to data collection in Canada, systemic inequities in our society will remain invisible.

Identifying racial disparities must be accompanied by investigations into targeted interventions and implementation strategies that can address these inequities (Krieger & Davey Smith, 2016; VanderWeele & Robinson, 2014). Collecting data on race or any social determinants of inequity without grounding in Critical Race Theory and the promotion of structural change can perpetuate inequity (Ontario Human Rights Commission, 2022). Critical Race Theory is the intellectual and social movement that recognizes racism is embedded within laws, policies, and institutions, examines the ways race intersects with other forms of social stratification (e.g., class, gender), and aims to challenge and transform societal structures that perpetuate systemic racism (Crenshaw et al., 1995). Centuries of scholarship on the social creation of racial categories confirm that race, per se, is not the exposure or “cause” of disparities in health outcomes between racial groups (Krieger, 2014). Health equity advocates emphasize that studies of health disparities should consider the multiple dimensions of structural racism as fundamental causes (Bailey et al., 2021; Krieger, 2014; Krieger & Davey Smith, 2016). Structural racism and the embodiment of systematic racialization (ecosocial theory) (Krieger, 2014; Krieger & Davey Smith, 2016) are likely intermediates in the causal pathway between race and maternal and neonatal morbidity and mortality (VanderWeele & Robinson, 2014). For example, pregnant individuals of different races may experience different rates of pregnancy-related morbidity and mortality due to different rates of pre-existing medical conditions (e.g., chronic hypertension) or differential access to or provision of high-quality antenatal or obstetric care (Creanga et al., 2014a, 2014b) Another explanation could be that inequitable living and working conditions due to racism create stressors that result in inequitable mental and physical health outcomes (Callaghan et al., 2014; Krieger & Davey Smith, 2016).

In future, national data on Black communities that include additional information on other aspects of ethnicity (e.g., country of origin, immigrant and refugee status, primary language) will enable a nuanced understanding of Black health in Canada (Cénat, 2022). It is important to acknowledge that the Black population in Canada is not homogeneous, but comprises diverse communities with distinct cultural, geographic, and historical backgrounds. These include, for example, individuals of Afro-Caribbean, African, and Black Canadian descent, among others. Each group may experience racism and structural inequities differently, shaped by intersecting factors such as immigration status, language, socioeconomic position, and gender. Recognizing this diversity is essential for understanding the full spectrum of how anti-Black racism operates and for co-designing interventions that are responsive to the needs of different Black communities. Studies considering these distinctions and interrelated factors will help to elucidate the impacts of longstanding structural racism and social exclusion on the health of racialized peoples in Canada, promote cultural humility in maternity care, and inform evidence-based, clinical, and health policy targets to reduce racial disparities in maternal and neonatal health. A national plan for harmonized data gathering on race and other important social determinants of health in administrative and clinical databases is a necessary first step to realise this vision.

## Conclusion

Research on Black-White inequities in maternal and neonatal health in Canada is lacking. Limited race-disaggregated data in Canada has impeded efforts to understand and address disparities in maternal and neonatal health outcomes among Black and White individuals, contributing to continued loss of life, loss of potential, and diminished quality of life. Although the literature on the topic is sparse, a Black-White disparity in maternal and neonatal health in Canada is apparent, and a better understanding of the factors driving these differences is warranted. Conscious, equity-focused, and patient-centred race-based data collection can provide the impetus that clinicians, researchers, policymakers, and funding organizations need to support policies to break down structural and systemic barriers to health experienced by the Black communities.

## Contributions to knowledge

What does this study add to existing knowledge?

- This review highlights the presence of Black-White disparities in maternal and neonatal health in Canada and the lack of epidemiologic studies available to better understand these disparities.
- Black individuals were found to have higher rates of preeclampsia, gestational diabetes, and inadequate gestational weight gain, while infants born to Black individuals had higher rates of spontaneous fetal loss, perinatal mortality, preterm birth, small for gestational age infants, low Apgar scores, congenital heart disease, and admission to the neonatal intensive care unit.

What are the key implications for public health interventions, practice, or policy?

- By identifying the gaps in current research, this review emphasizes the need for national, population-based data to comprehensively examine racial disparities and the underlying factors contributing to these differences.
- Race-disaggregated data collection must be underpinned by a commitment to dismantling racism and in partnerships with racialized community members and in alignment with the EGAP (Engagement, Governance, Access, Protection) Framework.
- This work lays the foundation for more rigorous, equity-focused epidemiologic investigations in the Canadian setting.

## Supplementary Information

Below is the link to the electronic supplementary material.

Supplementary file1 (DOCX 157 KB)

## Data Availability

All review data are publicly available.

## Declarations

### Patient and public involvement

Several members of our authorship team are Black individuals that have experienced childbirth in Canada. One co-author is the Executive Director of MinoCare, which supports and provides access to an integrated community of anti-racist, culturally safe healthcare services and providers. This work was developed with engagement from the Black Reproductive Health Working Group, an Ontario, Canada-based organization of Black midwives, doulas, physicians, and other allied health professionals.
