# Supplementary material for: Maternal and neonatal health in Canada’s Black communities: A scoping review of epidemiologic studies
Source: Can J Public Health. 2025 Sep 4;117(3):442–53. doi: 10.17269/s41997-025-01102-9 (PMC13338109; doi:10.17269/s41997-025-01102-9)

**Supplementary information**

Appendix 1. OVID Embase search strategy
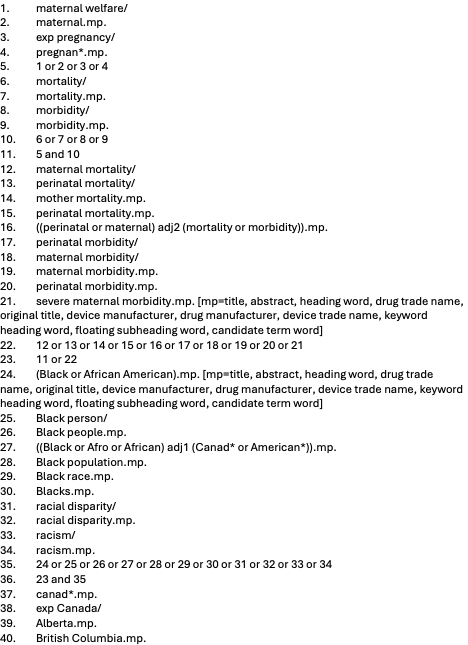


Appendix 2. OVID Medline search strategy


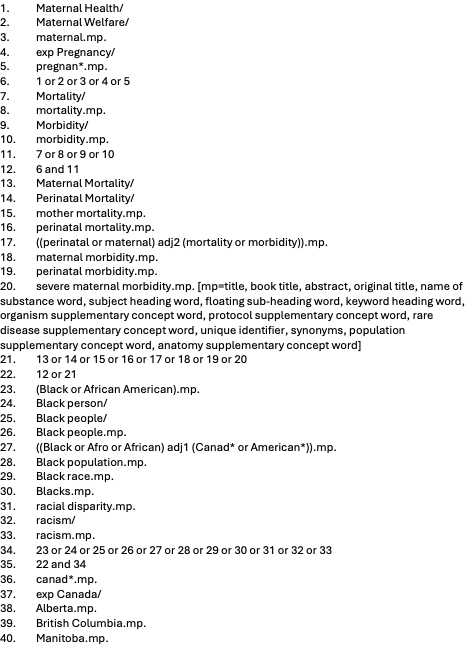


Appendix 3. OVID Emcare search strategy


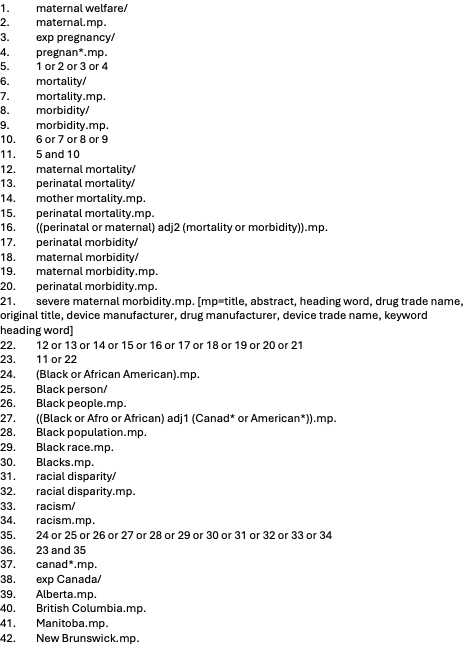

Supplement: Supplementary file 1 — Supplementary file1 (DOCX 157 KB) [file 41997_2025_1102_MOESM1_ESM.docx]
